# Evaluation of Histone Deacetylases as Drug Targets in Huntington’s Disease models: Study of HDACs in brain tissues from R6/2 and CAG140 knock-in HD mouse models and human patients and in a neuronal HD cell model.

**DOI:** 10.1371/currents.RRN1172

**Published:** 2010-09-02

**Authors:** Luisa Quinti, Vanita Chopra, Dante Rotili, Sergio Valente, Allison Amore, Gianluigi Franci, Sarah Meade, Marta Valenza, Lucia Altucci, Michele M. Maxwell, Elena Cattaneo, Steven Hersch, Antonello Mai, Aleksey Kazantsev

**Affiliations:** ^*^MassGeneral Institute for Neurodegenerative Disease, Massachusetts General Hospital, & Harvard Medical School, Bldg. 114-3300, 16th Street, Charlestown, MA 02129, USA; ^†^MassGeneral Institute for Neurodegenerative Disease, Massachusetts General Hospital, & Harvard Medical School, Bldg. 114-2350, 16th Street, Charlestown, MA 02129, USA; ^‡^Department of Chemistry and Technologies of Drugs Sapienza University of Rome; ^§^Istituto Pasteur-Fondazione Cenci Bolognetti, Dipartimento di Chimica e Tecnologie del Farmaco, Sapienza Università di Roma; ^#^General pathology department of Second University of Naples, via L. De Crecchio n° 7 CAP 80138, Naples; & Institute of Genetics and Biophysics CNR, via Pietro Castellino n°111 CAP 80131, Naples.; ^††^University of Milan; ^§§^Massachusetts General Hospital and Harvard Medical School, Boston, Massachusetts, USA; ^¶¶^Department of Pharmacological Sciences and Centre of Stem Cell Research, University of Milan; ^##^Massachusetts General Hospital/Harvard Medical School; Universita di Roma and Harvard Medical School/Massachusetts General Hospital

## Abstract

The family of histone deacetylases (HDACs) has recently emerged as important drug targets for treatment of slow progressive neurodegenerative disorders, including Huntington’s disease (HD). Broad pharmaceutical inhibition of HDACs has shown neuroprotective effects in various HD models. Here we examined the susceptibility of HDAC targets for drug treatment in affected brain areas during HD progression. We observed increased HDAC1 and decreased HDAC4, 5 and 6 levels, correlating with disease progression, in cortices and striata of HD R6/2 mice. However, there were no significant changes in HDAC protein levels, assessed in an age-dependent manner, in HD knock-in CAG140 mice and we did not observe significant changes in HDAC1 levels in human HD brains. We further assessed acetylation levels of α-tubulin, as a biomarker of HDAC6 activity, and found it unchanged in cortices from R6/2, knock-in, and human subjects at all disease stages. Inhibition of deacetylase activities was identical in cortical extracts from R6/2 and wild-type mice treated with a class II-selective HDAC inhibitor. Lastly, treatment with class I- and II-selective HDAC inhibitors showed similar responses in HD and wild-type rat striatal cells. In conclusion, our results show that class I and class II HDAC targets are present and accessible for chronic drug treatment during HD progression and provide impetus for therapeutic development of brain-permeable class- or isoform-selective inhibitors.

## Introduction 

      The HDAC family includes eleven Zn^++^-dependent deacetylases belonging to three structural classes [1] [2]. HDAC class I and class II deacetylases share significant structural homology, especially within the highly conserved catalytic domains. Ubiquitously expressed class I HDACs 1, 2, and 3 are components of stable transcriptional repressor complexes, involved in global transcriptional regulation. Class II includes HDAC4, HDAC5, HDAC7 and HDAC9, exhibiting distinct tissue-specific patterns of expression, and the ubiquitously expressed cytoplasmic microtubule (α-tubulin) deacetylase, HDAC6.

      In the past years numerous studies have demonstrated neuroprotection by small molecule HDAC inhibitors, broadly modulating all HDAC enzymes, in various human disease models, including Huntington’s disease (HD) [3] [4] [5]. HD is an autosomal-dominant neurodegenerative disorder, caused by the expansion of a CAG-triplet repeat within the coding sequences of the HD gene, IT15 [6]. The mutant huntingtin protein, containing a pathologically expanded polyglutamine sequence near the N-terminus, causes a progressive and fatal neurological phenotype [7]. Dysfunction and degeneration of cerebral cortical and striatal neurons underlie the symptoms of HD and the progressive functional decline that occurs [8] [9]. The precise mechanism(s) of neurodegeneration remain unknown, however neuronal homeostasis is profoundly perturbed by transcriptional dysregulation, abnormal histone acetylation and chromatin remodeling, aberrant protein interactions, mutant protein misfolding and aggregation, defects in axonal transport, and synaptic dysfunction. 

      Alterations in transcriptional regulation have been proposed to be especially significant for HD [10] [11] [12] [13], and HDAC inhibition has been suggested as a therapeutic strategy for modulating transcriptional pathology. The neuroprotective effects of HDAC inhibition have been well-documented in both invertebrate and mouse models of HD [3] [5] [14] [15] [16]. The involvent of HDACs in HD is shown in Table 1. The observed benefits have been attributed to a general HDAC-mediated chromatin remodeling and amelioration of transcriptional dysregulation by targeting HDACs 1-3 [17]. Alternatively, efficacy could be related to specific HDAC targets, as suggested by experimental data in invertebrate HD models. In *C. elegans* it has been shown that HDAC3 (HAD-3), but not HDAC1 (HAD-1), modulates polyglutamine-associated toxicity [18], whereas in *Drosophila*, neuroprotection was achieved by specific knockdown of rpd3 (the fly ortholog of HDAC1/2) [19].

**Table 1: Therapeutic effects of inhibiting HDAC isoforms in HD models.**

**Table:** 

**HDAC**	** Function(s)**	** Effect in HD Model**	** Ref.**
Class I, HDAC1	Global regulation of transcription	Neuroprotective in fly HD model	[19]
Class I, HDAC2	Global regulation of transcription	Neuroprotective in fly HD model	[19]
Class I, HDAC3	Global regulation of transcription	Neuroprotective in *C.elegans* HD model	[18]
Class IIa, HDAC4	Transcriptional repression	n.d.	
Class IIa, HDAC5	Transcriptional repression	n.d.	
Class IIa, HDAC7	Transcriptional repression	No effect in R6/2 crossed with HDAC7 knock-out mice	[20]
Class IIb, HDAC6	Microtubule transport, autophagy	Ameliorates microtubule transport defect, increases BDNF release in HD neurons *in vitro*	[21]

      It has also been demonstrated that inhibition of the activity of the class IIb deacetylase HDAC6 may compensate for the microtubule-dependent transport deficit in HD by increasing α-tubulin acetylation [21]. Further, recent results implicate HDAC6 as a regulatory protein for the major cellular protein degradation pathways: the ubiquitin-proteasome system and autophagy [22], both of which are linked with aggregation and clearance of mutant huntingtin. 

      Since HD is an age-dependent and slowly progressive disorder, neuroprotective therapy would require chronic drug treatment. Thus, in this study we have assessed, in an age-dependent manner, the presence and availability of HDAC targets for pharmacological treatment.

## Results

      First, we examined levels of class I HDAC proteins at different stages of disease progression in the transgenic R6/2 mouse model of HD. In R6/2 mice high expression levels of a mutant huntingtin exon I fragment containing ~160 CAG repeats lead to early neurological phenotype and premature death at 110-120 days [7] We assessed expression levels of HDAC1 and HDAC3 proteins in cortex and striatum, the primary brain areas affected by HD, of R6/2 transgenic mice and wild-type littermates. HDAC profiling in cortex was performed on mice sacrificed at 4 and 12 weeks of age, and in striatal samples on animals sacrificed at 9 weeks of age. Increased levels of HDAC1 protein in cortical samples from R6/2 mice were observed at both time points (**Fig. 1 A-C**). Similarly, in the striatum from 9 week-old R6/2 mice, levels of HDAC1 were elevated (**Fig. 1 D, E**). 

**Figure:**
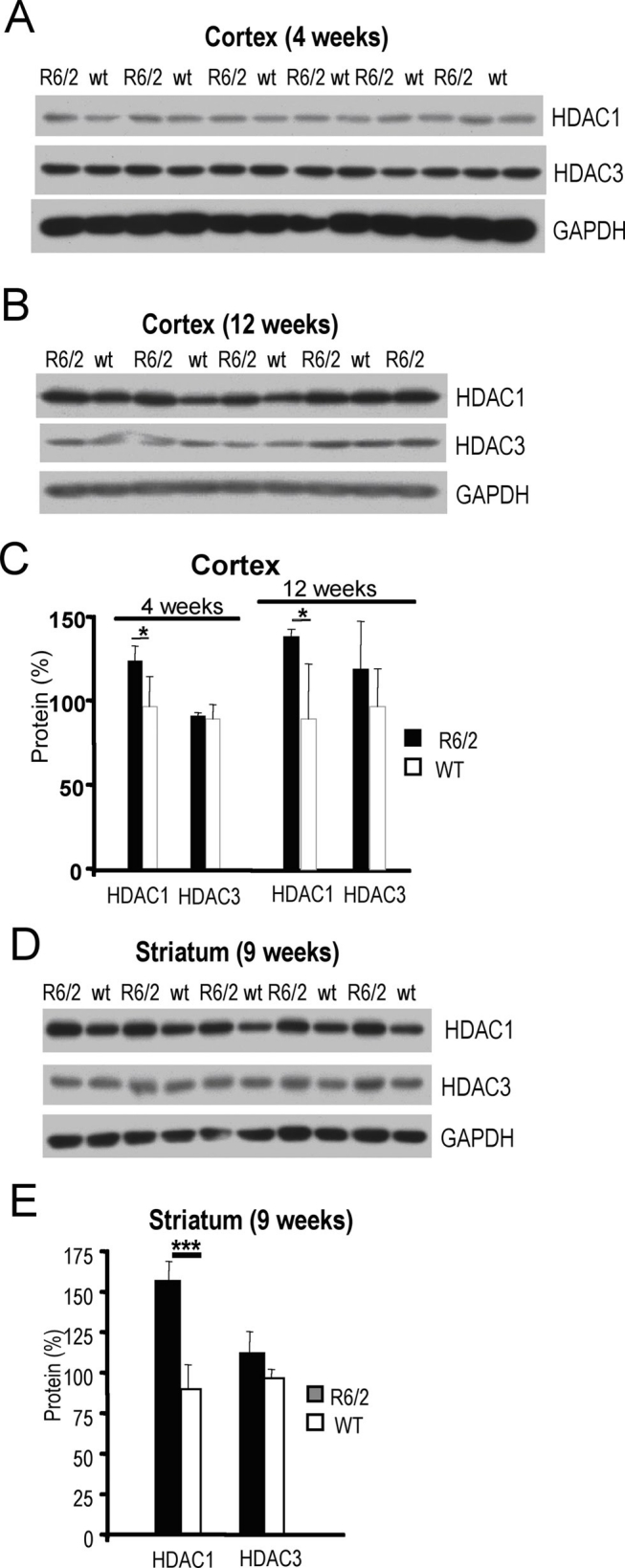
**Figure 1.** **Levels of class I HDAC proteins in cortex and striatum of R6/2 HD mouse model.** **A**) Western blot showing class I HDAC1 and 3 protein levels in cortices of 4 week-old R6/2 transgenic (R6/2) and wild-type (wt) mice. **B**) Western blot showing class I HDAC1 and 3 protein levels in cortices of 12 week-old R6/2 transgenic (R6/2) and wild-type (wt) mice. GAPDH was used as control in (**A-B**). **C**) Quantification of class I HDAC protein levels shown in (**A-B**). Black and white bars represent levels in R6/2 and in wild-type mice, respectively. **D**) Western blot showing class HDAC1 and 3 protein levels in striata of 9 week-old R6/2 transgenic (R6/2) and wild-type (wt) mice. GAPDH was used as control. **E**) Quantification of class I HDAC protein levels shown in (**D)**. Black and white bars represent levels in R6/2 and in wild-type mice, respectively. Statistical analyses were performed using the Student’s t-Test: * P<0.05; ** P<0.01; ***P<001. 

Next, we repeated profiling of HDAC class I proteins in CAG140 full-length knock-in HD mice. The CAG140 knock-in mouse model is genetically more representative of the human disease and has analogous neuropathology, but these mice develop only a modest neurological phenotype during their lifespan [23]. We analyzed levels of class I HDAC proteins in cortices from young (8 month-old) and aged (24 month-old) CAG140 knock-in mice and their wild-type littermates. The levels of HDAC1, HDAC2 and HDAC3 in CAG140 knock-in animals at 8 and at 24 months of age were unchanged (**Fig. 2A-C**). 

**Figure:**
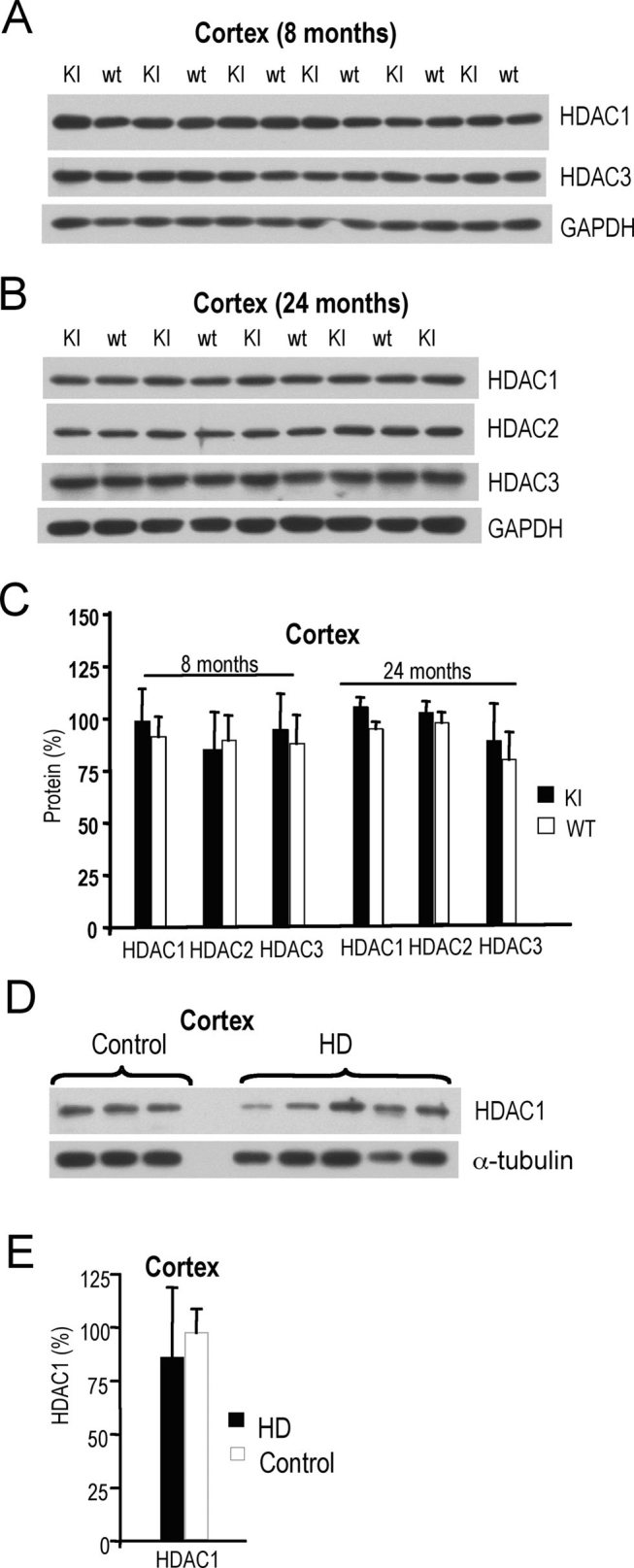
**Figure 2. Levels of class I HDAC proteins in cortex of CAG140 knock-in mice and HD patients.** **A**) Class I HDAC1 and 3 protein levels in cortices of 8 month-old CAG140 knock-in (KI) and wild-type (wt) mice detected by western. **B**) Class I HDAC1, HDAC2, and HDAC3 protein levels in cortices of 24 month-old CAG140 knock-in (KI) and wild-type (wt) mice. GAPDH was used as control in (**A-B**). **C**) Quantification of class I HDAC protein levels from western blots shown in (**A-B**). **D**)Class I HDAC1 protein levels in cortices of HD patients and normal subjects (control). α-Tubulin was used as control. **E**) Quantification of class I HDAC1 protein levels shown in (**D**). Statistical analyses were performed using the Student’s t-Test. * P<0.05; ** P<0.01; ***P<001. 

When we assessed the levels of HDAC1 in cortical samples from HD patients and normal individuals (**Table** **2**), HDAC1 levels appeared to be the same (**Fig. 2 D, E**). However, samples from HD patients and normal subjects were not controlled for disease grade or age. We also observed high variability of HDAC1 levels in human HD samples.

**Table 2: Required case information for the human brain tissues analyzed.**

**Table:** 

**Brain ID**	** NPDX**	** Sex**	** Age**	** Pmi (hrs)**
1400	HD	F	53	8
48	HD	F	61	6.3
1498	HD	M	48	12
1521	HD	M	49	18
77	HD	M	69	39
169	Control	F	82	20
171	Control	F	57	9
176	Control	M	41	7
161	Control	M	49	15

      Next, we examined levels of HDAC class II proteins at different stages of HD in the mouse models. There were no pronounced differences at the early stage of disease in R6/2 mice (4 weeks) **(Fig. 3A, C)**. However, at end-stage disease in R6/2 mice (12 weeks) we observed statistically significant reductions in levels of class II HDAC proteins, particularly HDAC6 (**Fig. 3B, C)**. Reduced HDAC class II protein levels were not limited to cortex, but were also observed in striata from 9 week-old R6/2 mice (**Fig. 3D, E)**.

**Figure:**
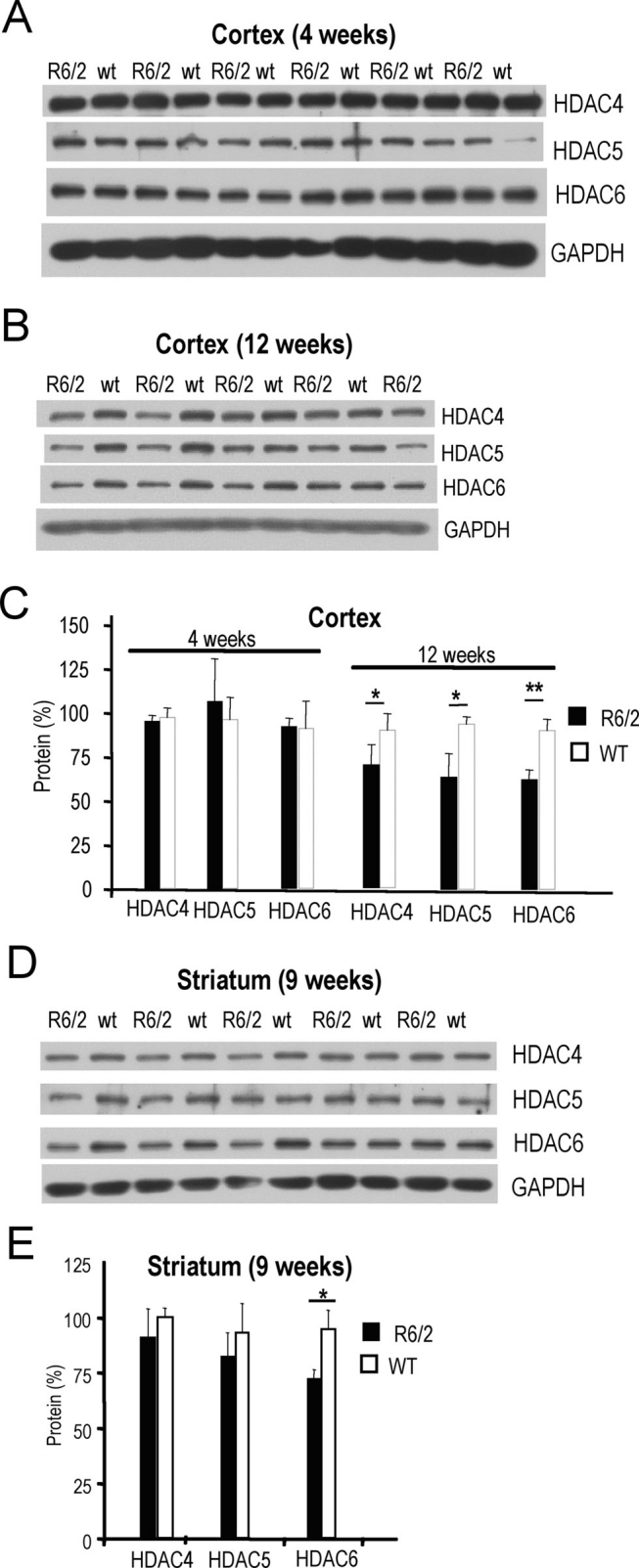
**Figure 3**. **Levels of HDAC4, HDAC5, and HDAC6 proteins** **in cortex and striatum of R6/2 HD mice**. **A) **Western blot showing HDAC4, HDAC5 and HDAC6 protein levels in cortices of 4 week-old R6/2 transgenic (R6/2) and wild-type (wt) mice. **B) **HDAC4, HDAC5, and HDAC6 protein levels in cortices of 12 week-old R6/2 transgenic (R6/2) and wild-type (wt) mice. GAPDH was used as control in (**A-B**). **C**) Quantification of class II HDAC protein levels from westerns shown in (**A-B)**. Black and white bars represent levels in R6/2 and in wild-type mice, respectively. **D**) Class II HDAC4, 5, 6 protein levels in striatum of 9 week-old R6/2 transgenic (R6/2) and wild-type (wt) mice. GAPDH was used as control. **E**) Quantification of class II HDAC protein levels from western blots shown in (**D)**. Black and white bars represent levels in R6/2 and in wild-type mice, respectively. Statistical analyses were performed using the Student’s t-Test. * P<0.05; ** P<0.01; ***P<001. 

      In the HD knock-in CAG140 mouse model, we did not observe significant differences in the levels of HDAC 4, 5 and 6 proteins at early (8 month-old) or late (24 month-old) disease stage (**Fig. 4**). High variability of HDAC5 and HDAC6 protein levels in HD and wild-type aged mice were observed. Similarly, in the human samples, the high variability of HDAC class II proteins did not permit us to detect any clear difference in HD *vs.* control samples (**not shown**).

**Figure:**
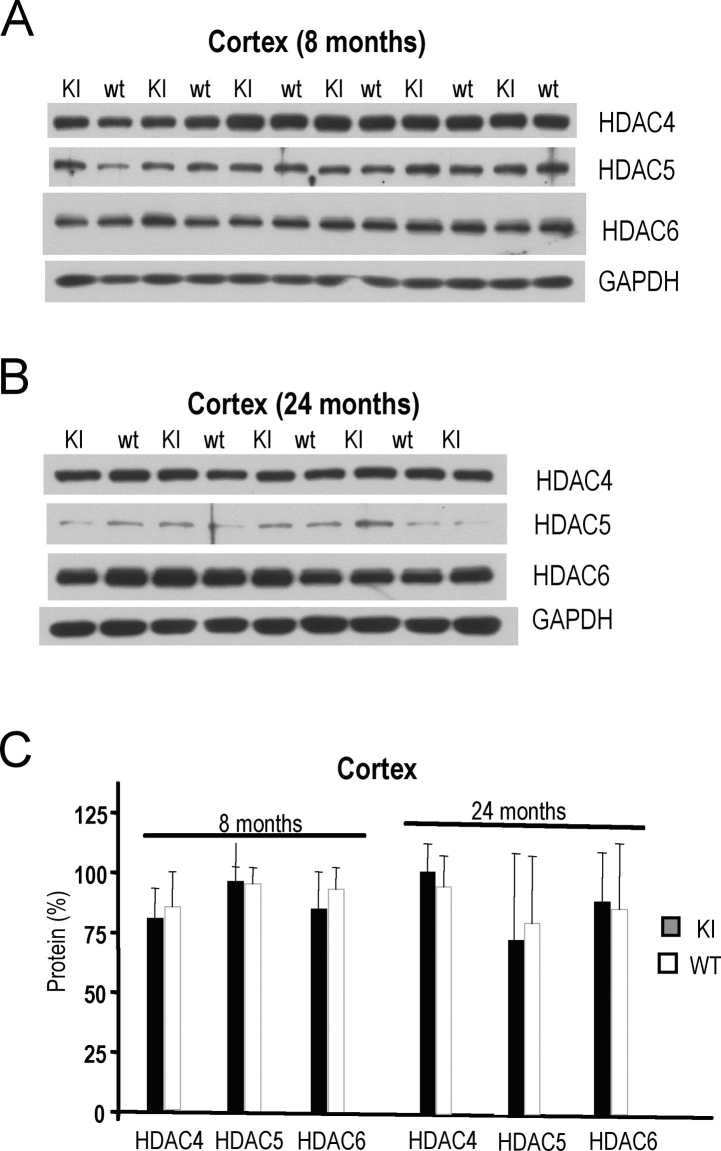
**Figure 4. Levels of class II HDAC4, HDAC5, HDAC6 proteins in cortex of CAG140 HD knock-in mice. A**) Western blot showing class II HDAC protein levels in cortices of 8 month-old CAG140 knock-in (KI) and wild-type (wt) mice. **B**) Class II HDAC protein levels in cortices of 24 month-old CAG140 knock-in (KI) and wild-type (wt) mice. GAPDH was used as control in (**A-B**). **C**) Quantification of class I HDAC protein levels from western blots shown in (**A-B**). 

       To overcome this hurdle we extended our analysis to assess the state of α-tubulin acetylation in HD mouse and human brains. Because α-tubulin is a substrate of the microtubule deacetylase HDAC6, acetylated α-tubulin levels function as a biochemical marker for HDAC6 cellular activity. We examined whether modulation of HDAC6 levels in R6/2 cortices (**Fig. 5A, Fig. 6** **A**) and striata (**Fig.** **6** **B**) are translated into higher levels of α-tubulin acetylation. In parallel, we assessed α-tubulin acetylation levels in HD knock-in CAG140 mice (**Fig. 5B, Fig.** **6** **D**) and in human samples (**Fig. 5C**). We did not observe statistically significant changes in α-tubulin acetylation in any of these tissues (**Fig. 5D**). Lastly, we used a pharmacogenomic approach to compare the extent of deacetylase inhibition in cortical extracts from 12 week-old R6/2 and wild-type mice, by using a previously described class II selective inhibitor, MC1568, as a molecular probe [24] [25] [26] [27] [28] [29] [30] [31] [32]. This compound inhibited deacetylase activities in an identical manner in HD mutant and wild-type cortical samples (**Fig. 5E**). 

**Figure:**
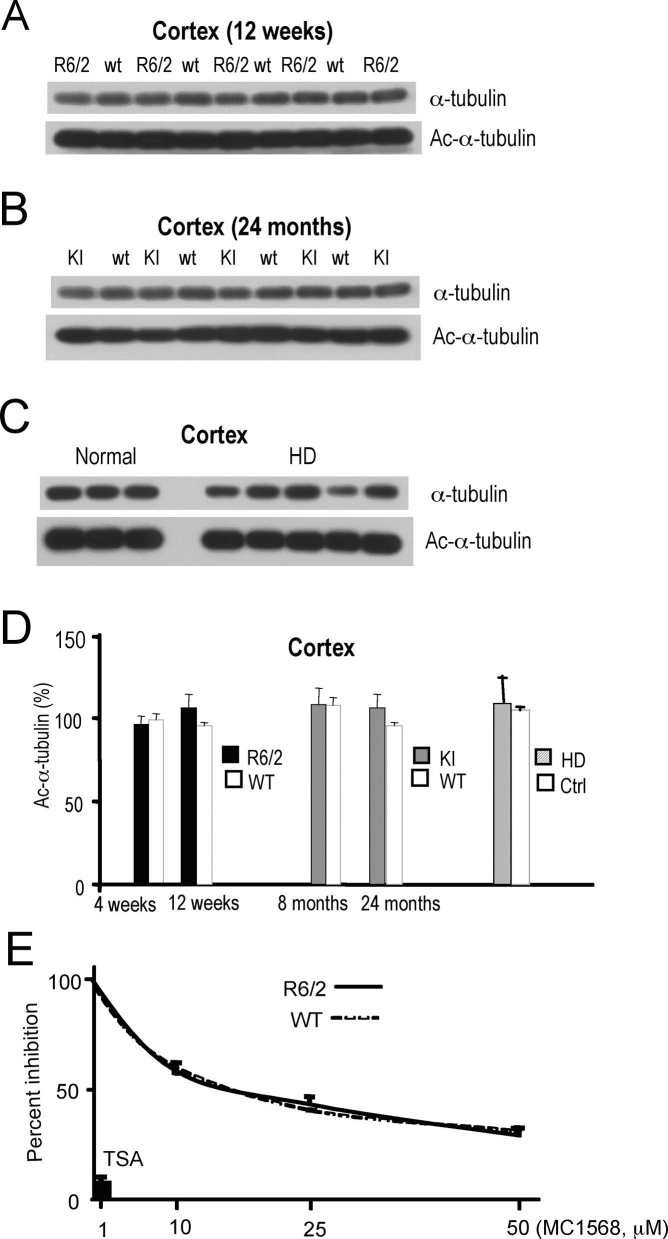
**Figure 5**. **Evaluation of acetylated** **a-tubulin levels in HD cortices.** **A-C**) Western analysis of acetylated and total a-tubulin in cortices: **A**) 12 week-old R6/2 transgenic (R6/2) and wild-type (wt) mice; **B**) 24 month-old CAG140 knock-in (KI) and wild-type mice; **C**) normal control and HD human samples. **D**) Quantification of α-tubulin acetylation normalized to total α-tubulin, from westerns shown in **A-C**). Black bars, R6/2; grey bars, KI; stripped grey bars, HD patients; white bars wild-type and normal controls, respectively.** E**) Dose-response inhibition of deacetylase activities in cortical extracts of 12 week-old R6/2 (solid line) and wild-type (dotted line) mice treated with the HDAC class II selective inhibitor MC1568. In vitro assays were conducted with exogenous fluorophor-containing acetylated substrates. Pan HDAC inhibitor TSA at 1 μM concentration was used as control. 

**Figure:**
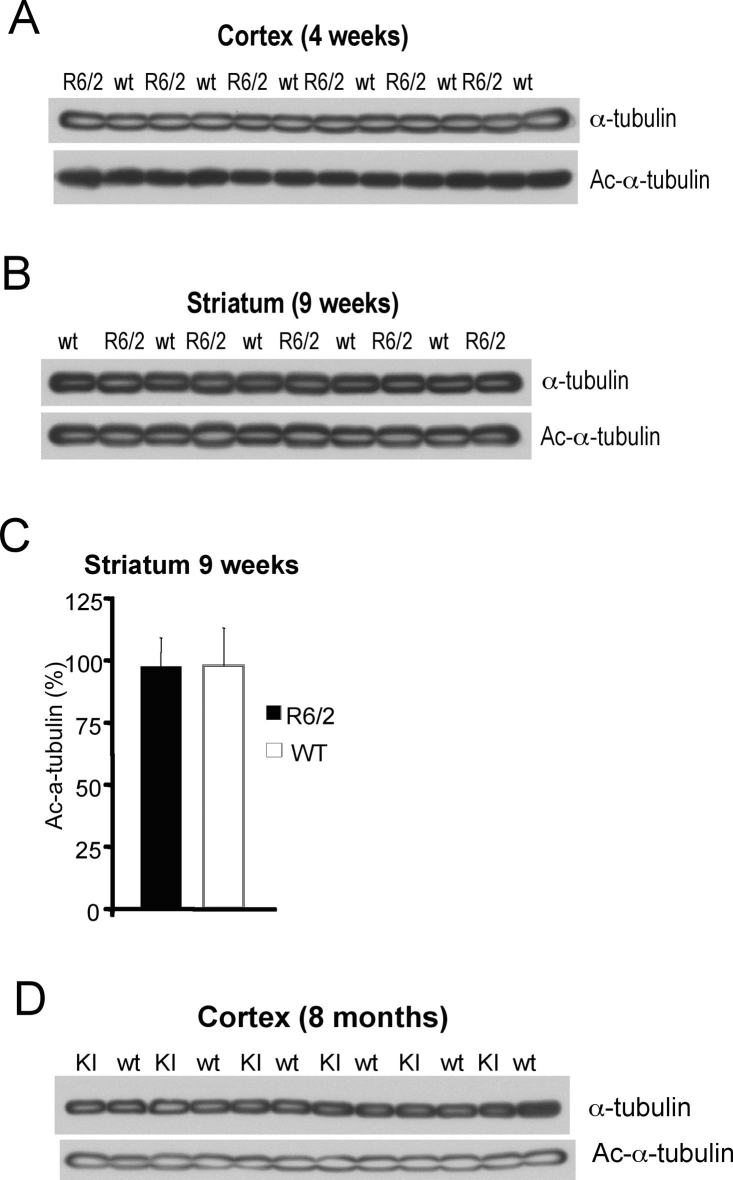
**Figure** **6. Evaluation of acetylated a-tubulin levels in HD.** **A**) Acetylated and total α-tubulin detected by western in cortices of 4 week-old R6/2 transgenic (R6/2) and wild-type (wt) mice; **B**) Acetylated and total α-tubulin detected by western in striata of 9 week-old R6/2 transgenic (R6/2) and wild-type (wt) mice; **C**) Quantification of α-tubulin acetylation normalized to total α-tubulin, shown in (**B**). R6/2 and wild-type mice represented by black and white bars, respectively. **D**) Acetylated and total a-tubulin in cortices of 8 month-old CAG140 knock-in (KI) and wild-type (wt) mice. 

Using a similar pharmacological approach, we then tested whether the presence of mutant huntingtin interfered with acetylation/deacetylation of HDAC substrates in a cellular environment physiologically relevant to HD. To that end we tested class-selective HDAC inhibitors in the previously characterized rat embryonic striatal ST14A cells, which express stably integrated mutant (128Q) or wild-type (15Q) 548 amino acid N-terminal fragments of human huntingtin [33] [34]. Compound experiments were conducted in cycling and in differentiated post-mitotic cells. Mutant HD cells demonstrated the expected increase in acetylation in response to treatment with HDAC class I selective MC2070 (A.Mai, S.Valente, M.Tardugno, M.Conte, R.Cirilli, A.Perrone, S.Massa, A.Nebbioso, G.Brosch and L.Altucci, manuscript in preparation) and with class II selective inhibitors MC1568 and MC1575 [24] [25] [26] [27] [28] [29] [30] [31] [32] (**Table** **3** **,** **4**). 

**Table 3:** **Chemical structures of tested compounds.** Structures of class II selective HDAC inhibitors MC1568 and MC1575 [24] [25] [26] [27] [28] [29] [30] [31] [32] and class I selective inhibitor MC2070 (A.Mai, S.Valente, M.Tardugno, M.Conte, R.Cirilli, A.Perrone, S.Massa, A.Nebbioso, G.Brosch and L.Altucci, manuscript in preparation).

**Figure:**
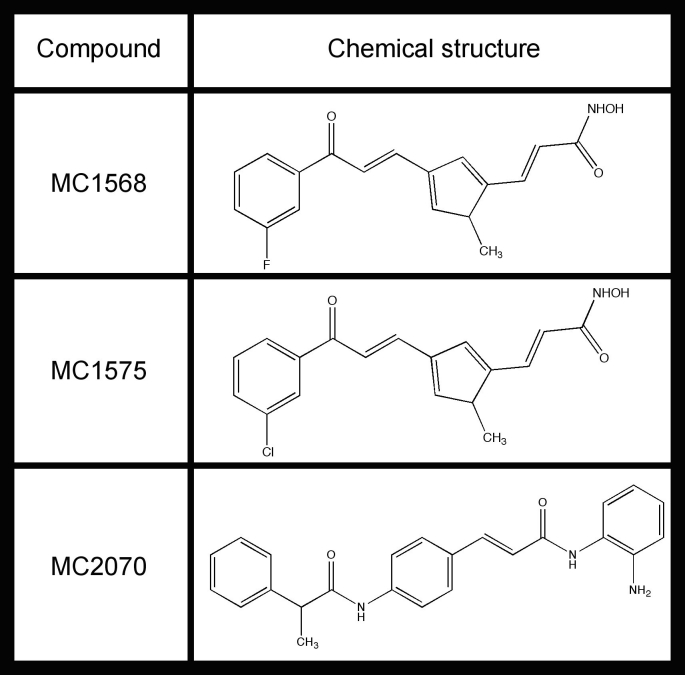

**Table 4: HDAC inhibition data of tested compounds.** Relative HDAC inhibition activities of tested compounds MC1568, MC1575 (class II HDAC selective), and MC2070 (class I HDAC selective), expressed as percent of control (100%). 

**Table:** 

	**HDAC1**	**HDAC2**	**HDAC3**	**HDAC4**	**HDAC5**	**HDAC6**	**HDAC8**
**MC1568 5μM**	93.5	98.1	102.8	57.0	34.6	75.9	87.6
**MC1575 5μM**	84.8	112.8	110.8	69.2	n.d.	68.3	87.7
**MC2070 5μM**	28.7	n.d.	n.d.	100.0	n.d.	n.d.	n.d.

In details, consistent with the inhibitors class-selectivity, MC2070 and MC1568 respectively increased histone and α-tubulin acetylation in HD treated cells (**Fig. 7A**). In wild-type cells the effects of the compounds were highly similar (**not shown**). We next performed side-by-side comparison, in HD mutant and wild-type cells, of the class II selective inhibitor MC1575 effects on α-tubulin acetylation (**Fig. 7B**). Acetylation levels were determined by western analysis of extracts from cells treated with the compound for 4 hours. Acetylation of α-tubulin increased at the same rate in mutant and wild-type cells, as indicated by quantification of the western blots (**Fig. 7C**).

**Figure:**
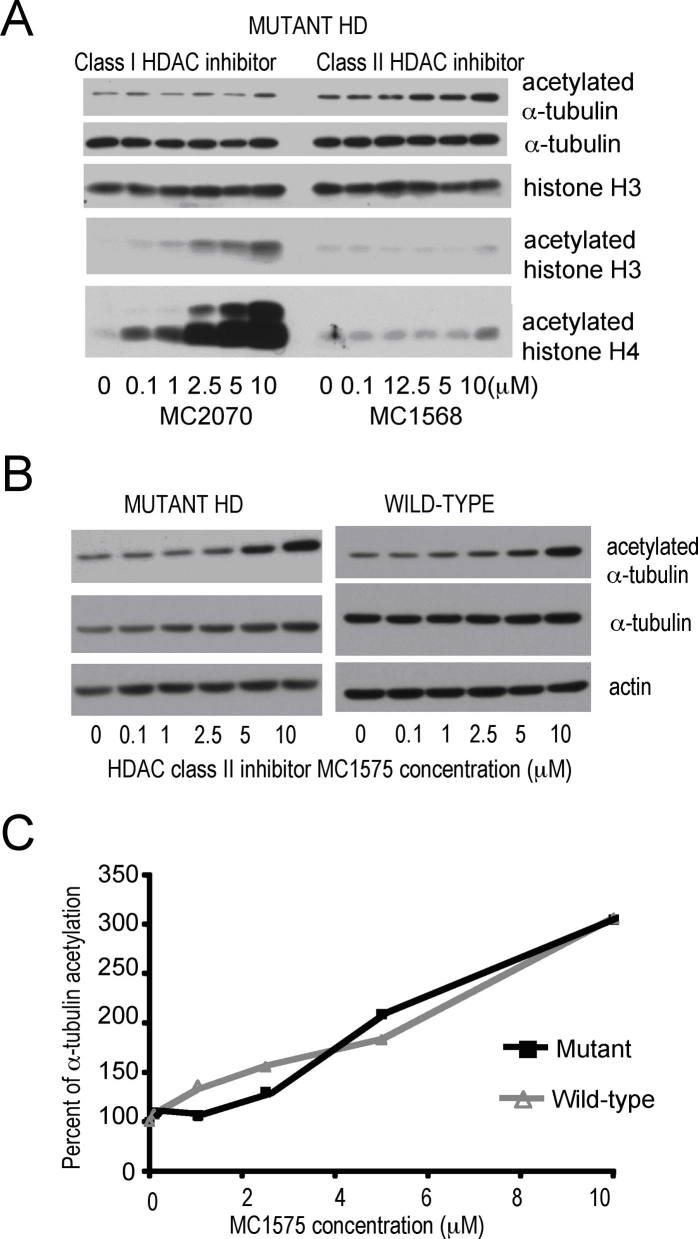
**Figure 7**. **Responses of HD cells to HDAC class-selective inhibition with small molecular probes.** **A**) Deacetylase activities inhibition in ST14A HD striatal cells with class I selective MC2070 and class II selective MC1568 inhibitors. Inhibitor effects on acetylation levels of class I substrates histones H3, H4 and class II substrate α-tubulin were detected by western blotting. **B**) Western blot showing dose-dependent effects of class II HDAC inhibitor MC1575 on acetylation levels of α-tubulin protein in mutant and wild-type ST14A cells. Actin was used as control. **C**) Dose-response curves of compound effects on α-tubulin acetylation in mutant HD (black squares and line) and wild-type (grey squares and line), based on quantification of western blots in (**B**). 

## Discussion 

      Our results demonstrate a statistically significant increase in HDAC1 and decrease in HDAC4, HDAC5 and HDAC6 levels in cortex and striatum of R6/2 transgenic HD mice at both early and terminal disease stages. The observed changes of HDAC class II protein levels in the R6/2 model correlate with disease progression. However, the extent to which these changes are components of neurodegeneration, of compensatory response(s), or are benign phenomena, remains to be determined. 

      In age-controlled experiments with CAG140 knock-in and wild-type mice, changes in levels of HDAC proteins were not statistically significant, although levels of HDAC5 and HDAC6 were too variable to permit a conclusion. It is noteworthy that the CAG140 knock-in mice display only a mild neurological HD phenotype as compared to R6/2 transgenic mice; this difference in phenotype may explain the discrepancy between our results in these two HD mouse models. Further, our study was largely based on evaluation of HDAC protein levels by western blotting; although this method was sufficiently sensitive to allow us to measure differences of >15-20% in R6/2 animals, it might not have permitted detection of more subtle changes in expression levels over time.

      We also observed high variability of HDAC protein levels was observed in human samples, which were not controlled for disease grade and age. This hindered our ability to draw conclusions regarding overall HDAC proteins levels in human disease, although it appeared that HDAC1 levels were not increased in HD. 

      We reasoned that a ~30% increase in HDAC1 protein levels in R6/2 cortical and striatal tissues would be unlikely to cause a detectable decrease in global histone acetylation.  The native substrates of HDAC4 and HDAC5 deacetylases are unknown. However, α-tubulin acetylation can serve as a sensitive biomarker to detect changes of HDAC6 activity. Notably, in our experiments we detected no changes in levels of α-tubulin acetylation in HD animals, and furthermore, the extent of deacetylase inhibition in cortical extracts from R6/2 and wild-type mice treated with a HDAC class II selective inhibitor were identical. Further, compound experiments demonstrated highly similar responses in HD and wild-type cells, suggesting that deacetylase and acetylase activities are not compromised in mutant cells. 

      In contrast to their marked activity in invertebrate HD models, pan-HDAC inhibitors like SAHA (suberoylanilide hydroxamic acid) or phenyl butyrate have shown only modest efficacy in mouse models [3] [5] [15]. These data suggest that broad inhibition of HDACs, which are responsible for regulating multiple cellular pathways, causes adverse side-effects that counter the efficacy of these inhibitors. Selective targeting of HDAC modalities is expected to improve efficacy and limit toxicity. 

      The overall goal of this study was to determine the presence and availability of HDAC enzymes as drug targets in Huntington’s disease. Although our results did not implicate a particular HDAC isoform for therapeutic targeting, the data presented here demonstrate that all class I and class II HDACs are present in human HD and in HD model mice throughout the disease course, and highlight their activities as attractive targets for chronic drug treatment. Developing brain-permeable class- and isoform-selective inhibitors with low predicted toxicity is an important therapeutic goal for medicinal chemistry optimization, which in its initial thrust can employ the inhibitors tested in this study.   

## Material and methods

### Mice and human tissues

The R6/2 transgenic and CAG140 knock-in mice used in this study were maintained on a B6/CBA background by crossing male HD mice with B6/CBA F1 females. Brain tissues were collected at 4, 9 & 12 weeks of age from R6/2 mice and at 8 and 24 months from CAG140 mice (n=5/group). At each time point tissues were also collected from equal numbers of wild-type littermates. All animal experiments were carried out in accordance with the National Institutes of Health Guide for the Care and Use of Laboratory Animals and were approved by the local animal care committee.

Human cortex samples from HD and control patients were obtained from the Tissue Resource Center of the Alzheimer Disease Research Center (ADRC) at Massachusetts General Hospital and from the New York brain bank and used according to the hospitals’ regulations.Detailed case information is provided in Table 2.

### Protein extraction from tissues

Frozen cortical and striatal tissues from mice and frozen cortical tissues from human samples were homogenized in PBS containing Complete EDTA-free Protease Inhibitor Cocktail (Roche Applied Science, USA) and 1mM PMSF (phenylmethanesulfonylfluoride or phenylmethylsulfonyl fluoride), using a Kontes Pellet Pestle (Kimble/Kontes, USA). They were then sonicated with a Branson Sonifier (Branson Ultrasonic Corp., USA) and lysed overnight at 4ºC in 3X volume of 63mM Tris buffer pH 6.8, 2% SDS, 10% glycerol, 1mM DTT, Complete EDTA-free Protease Inhibitor Cocktail and 1mM PMSF.

Protein concentration in the lysates was determined with the BCA protein assay kit (Pierce, Thermo Scientific, USA) and the appropriate volume of each sample was diluted in 3X SDS sample buffer (New England BioLabs, USA) for SDS-PAGE analysis.

### Proteins SDS-PAGE and immunoblotting

Protein lysates, containing 3X SDS sample buffer, were boiled at 100ºC for 2 min and then subjected to SDS-PAGE. Proteins were transferred onto 0.2 μm Immobilon-P membrane (Millipore, USA) and then blocked with a 5% milk solution in PBS-Tween for 1h. 

Blots were probed overnight at 4ºC with primary antibodies against the proteins of choice in 5% milk solution in PBST. Antibodies against actin (A2066), α-tubulin (T6074) and Ac-α-tubulin (T6793) were purchased from Sigma (Sigma-Aldrich, USA). Antibodies against HDAC1 (sc-7872), HDAC2 (sc-7899), HDAC3 (sc-11417), HDAC4 (sc-11418) and HDAC5 (sc-11419) were purchased from Santa Cruz Biotechnology (Santa Cruz Biotechnology Inc., USA). Antibodies against GADPH (glyceraldehyde 3-phosphate dehydrogenase) (MAB374), Ac-H3 (06-599) and Ac-H4 (06-946) were purchased from Millipore (Millipore, USA). Antibodies against H3 (9715), H4 (2592) and HDAC6 (2162) were purchased from Cell Signaling Technology (Cell Signaling Technology Inc., USA). Antibodies were used at concentrations recommended by their manufacturers.

Secondary detection was performed incubating the blots with HRP-conjugated secondary antibodies in 3% milk solution in PBST for 2h at room temperature. Proteins were visualized using an ECL detection substrate (Pierce, Thermo Scientific, USA).

Protein levels were quantified by densitometry with the ImageJ software (NIH, USA), normalizing to actin or GAPDH levels.

Statistical analyses were performed using the Student’s t-Test and statistical significance was reported as follows: * P<0.05; ** P<0.01; ***P<001.

### Deacetylase activity assay in mouse tissue extracts 

Cortical samples from 12 week-old wild-type and transgenic R6/2 mice were used. Each sample was weighed and homogenized in 20 volumes of 50mM Tris-HCl pH 8.0, 137mM NaCl, 2.7mM KCl, 1mM MgCl_2_, 1mg/ml BSA and 10% glycerol. 

TSA (trichostatin A) and MC1568 were tested against deacetylase activities in wild and R6/2 cortical extracts in standard acetylation reactions (Enzo Life Sciences, USA) supplemented with fluor-containing deacetylation substrates for deacetylation and NAD^+^ (nicotinamide adenine dinucleotide). 

### Cell culture and treatment

Wild-type and mutant rat embryonic striatal ST14A cells, a generous gift of E. Cattaneo, were cultured as previously described [34]. For testing class I and class II HDAC inhibitor bioactivity, wild-type and mutant ST14A cells were plated into 6-well plates and grown at 33°C. Upon reaching 80% confluency, cells were switched to serum-free media with N2-supplement (Invitrogen, USA), and moved to 37°C; at the same time compounds were added at concentrations ranging from 0 to 10 μM. DMSO was used as control. After 6-18 h incubation with compounds, cells were harvested and lysed overnight at 4ºC in 63mM Tris buffer pH 6.8, 2% SDS, 10% glycerol, 1mM DTT, Complete EDTA-free Protease Inhibitor Cocktail and 1mM PMSF. Protein concentrations were determined using a BCA protein assay kit and the appropriate volume of each sample was diluted in 3X SDS sample buffer for SDS-PAGE analysis.

### Compounds 

The class-selective HDAC inhibitors used in this study (MC2070, class I-selective, and MC1568 and MC1575, class II-selective) have been prepared as described (A.Mai, S.Valente, M.Tardugno, M.Conte, R.Cirilli, A.Perrone, S.Massa, A.Nebbioso, G.Brosch and L.Altucci, manuscript in preparation) [24] [25] [26] [27] [28] [29] [30] [31] [32].

Class I and II HDAC inhibitors were dissolved in molecular biology grade dimethyl sulfoxide (DMSO) to 10mM stock concentration, aliquoted and stored at -80°C.  

## Funding information 

This work was supported by The Carmen Foundation; by Fondazione Roma; and by AIRC and ATLAS [HEALTH-F4-2009-221952]. 

## Competing interests 

The authors have declared that no competing interests exist. 

Correspondence should be addressed to Aleksey Kazantsev: akazantsev@partners.org  
